# Surgical Approaches to Supradiaphragmatic Segment of IVC and Right Atrium through Abdominal Cavity during Intravenous Tumor Thrombus Removal

**DOI:** 10.1155/2014/924269

**Published:** 2014-01-22

**Authors:** Dmytro Shchukin, Vladimir Lesovoy, Igor Garagatiy, Gennadiy Khareba, Redouane Hsaine

**Affiliations:** ^1^Department of Urology, Nephrology and Andrology, Kharkiv National Medical University, 195 Moskovskiy Avenue, Kharkiv 61037, Ukraine; ^2^Department of General, Pediatric and Oncological Urology, Kharkiv Medical Academy of Postgraduate Education, 195 Moskovskiy Avenue, Kharkiv 61037, Ukraine

## Abstract

*Objective*. The purpose of this study was to investigate safety and feasibility of some surgical approaches to the supradiaphragmatic inferior vena cava (IVC) and the right atrium through the diaphragm from the abdominal cavity. *Materials and Methods*. The material of the anatomical study included 35 fresh cadavers. Several options of surgical access to the supradiaphragmatic IVC were successively performed. Feasibility and risk level of each of the approaches were evaluated with the use of a special scale. *Results*. The isolation of the supradiaphragmatic IVC and cavoatrial junction was most easily performed via T-shaped or circular diaphragmotomy (grade “easy” was registered in 74.3% and 80% of patients, resp., compared to 31.4% for transverse diaphragmotomy and 40% for isolation of the IVC in the pericardial cavity). The risk analysis has demonstrated the highest safety level for T-shaped diaphragmotomy (grade “safe” was registered in 60% of cases). The intervention via transverse diaphragmotomy, circular diaphragmotomy, and IVC isolation in the pericardial cavity was graded as “risky” in 80%, 62.9%, and 82.9% of cases, respectively. *Conclusions*. In our opinion, T-shaped diaphragmotomy is the most safe and easy-to-perform access for mobilization of the supradiaphragmatic IVC through the abdominal cavity.

## 1. Introduction

One of the most important aspects of surgical treatment for renal tumors extending into the inferior vena cava is control of the distal end of the tumor thrombus. This stage presents a challenge due to “high” localization of the thrombus apex (the retrohepatic and intrapericardial IVC, the right atrium) and mainly depends on the type of approach selected. Traditionally, for these patients the techniques of cardiopulmonary bypass, hepatic vascular exclusion, and deep hypothermic circulatory arrest are used. To do this, in addition to laparotomy access it is necessary to perform either sternotomy or thoracotomy. However, this surgical technique results in wide opening of several body cavities, significantly increases the duration of surgery, and also can be accompanied by specific postoperative complications, such as mediastinitis, pain in wounds caused by sternotomy, pericardial adhesion scars, coagulopathy, and central nervous system complications [1].

In recent years there have been more and more reports on an alternative surgical approach without use of cardiopulmonary bypass and circulatory arrest [2]. For this purpose, the use of various surgical approaches to the supradiaphragmatic segment of the IVC and the right atrium solely through the abdominal cavity has been proposed [3–10]. However, the summarized experience of surgical interventions with the use of this approach is quite little. Besides, the anatomy of the supradiaphragmatic inferior vena cava in terms of thrombectomy is inadequately understood.

It is currently unclear whether it is possible to perform an adequate access to cavoatrial segment without opening the pericardium and if it is safer to apply the tourniquet around the intrapericardial IVC either through the pericardium or outside the pericardium. Another important issue to be considered is the risk of serious intraoperative (injury of the supradiaphragmatic IVC, the right phrenic nerve, or the phrenic veins) and postoperative (dysfunction of the diaphragm) complications when these types of access are used.

We have conducted an anatomical study for feasibility and safety of several options of surgical approaches to the supradiaphragmatic IVC and the right atrium through the diaphragm from the abdominal cavity.

## 2. Materials and Methods

In this anatomical study we used 35 fresh cadavers (less than 48 hours after death). The autopsies were carried out between June and September 2012 on the base of the morbid anatomy departments of Hospital Number 8 and V.I. Shapoval Regional Clinical Center of Urology and Nephrology in the city of Kharkov, Ukraine. The study was approved by the Local Ethics Committee for V.I. Shapoval Regional Clinical Center of Urology and Nephrology (Protocol No. 3 dated May 17, 2012). The age of the deceased patients (18 males and 17 females) ranged from 42 to 85 years old and averaged 69.3 years. The average height did not exceed 168 cm, and the weight was not more than 82 kg.

To examine the aspects of intrapericardial IVC and its tributaries the following technique was used. After removal of the organs by en masse technique, the posterior surface of the entire length of inferior vena cava was sharply and bluntly exposed. Thereafter, target evaluation of the topography and size of the identified phrenic veins as well as the right phrenic nerve and its branches was performed. After the organs ventral side was turned up the mobilization of the liver was performed using the classical method (intersection of the falciform, triangular and coronary ligaments), which enabled us to locate the suprahepatic infradiaphragmatic IVC with the insertions of the major hepatic veins, the zone of the IVC passing through the tendon center of the diaphragm, and to evaluate the topography of the phrenic vein insertions in this area. A vascular tourniquet was placed around the suprahepatic subphrenic segment of the inferior vena cava, after that followed successive performance of several options of surgical access to the supraphrenic IVC without opening the pericardium: transverse, T-shaped, and circular diaphragmotomy.

**Figure 1:**
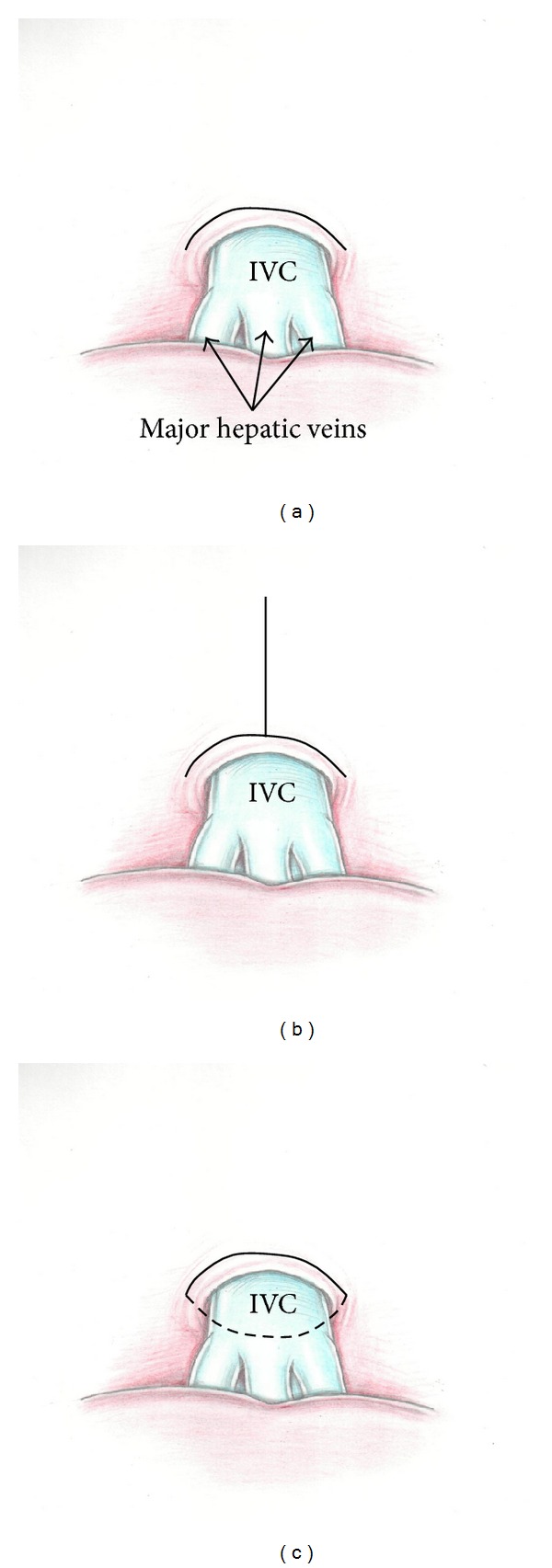
Types of diaphragmotomy: ((a) transverse diaphragmotomy, (b) T-shaped diaphragmotomy, and (c) circular diaphragmotomy).

Transverse diaphragmotomy included an incision of the diaphragm parallel to the anterior half circle of the inferior vena cava with the margin of 3–5 mm. T-shaped diaphragmotomy presented a similar incision in the diaphragm, added with a perpendicular longitudinal incision within 3-4 cm. Circular diaphragmotomy involved total circular isolation of the IVC from the diaphragm with 3–5 mm margin of the anterior vena cava and with 1-2 mm margin of the posterior vena cava (Figure 1).

**Figure 2:**
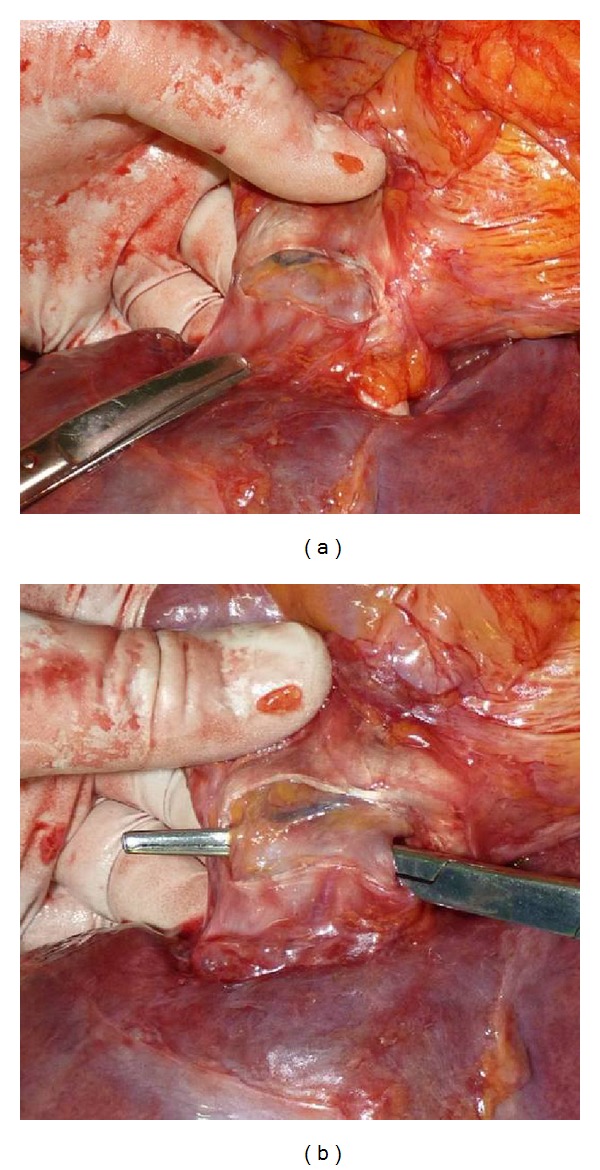
(a) Transverse diaphragmotomy; (b) surgical clamp passed posterior to the supradiaphragmatic IVC.

**Figure 3:**
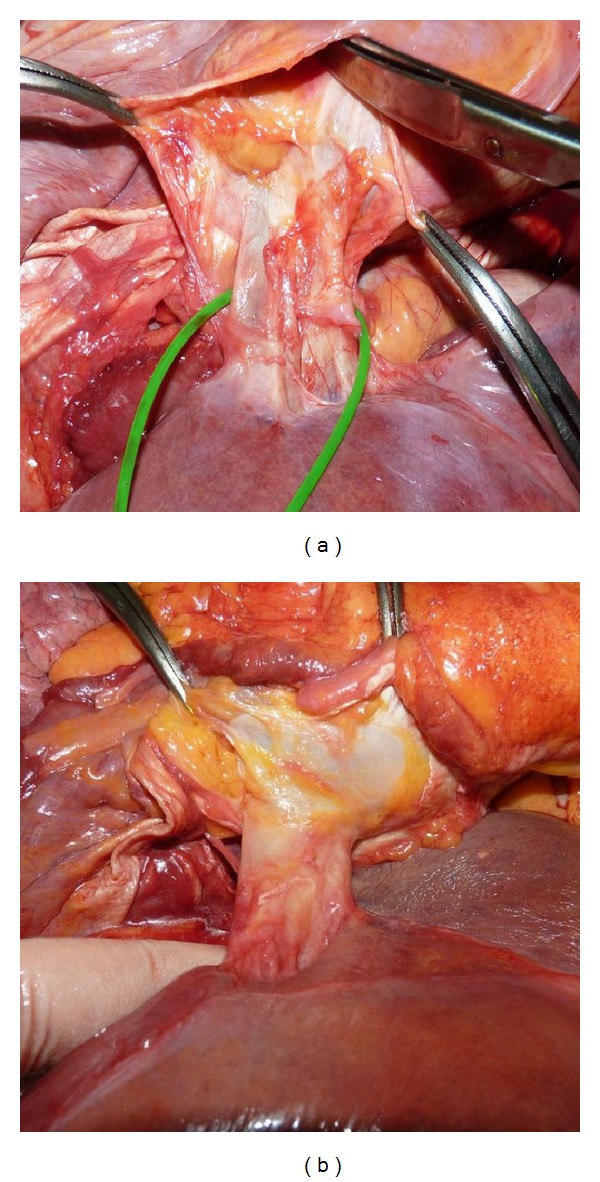
(a) T-shaped diaphragmotomy; (b) right atrium covered with pericardium after T-shaped diaphragmotomy.

**Figure 4:**
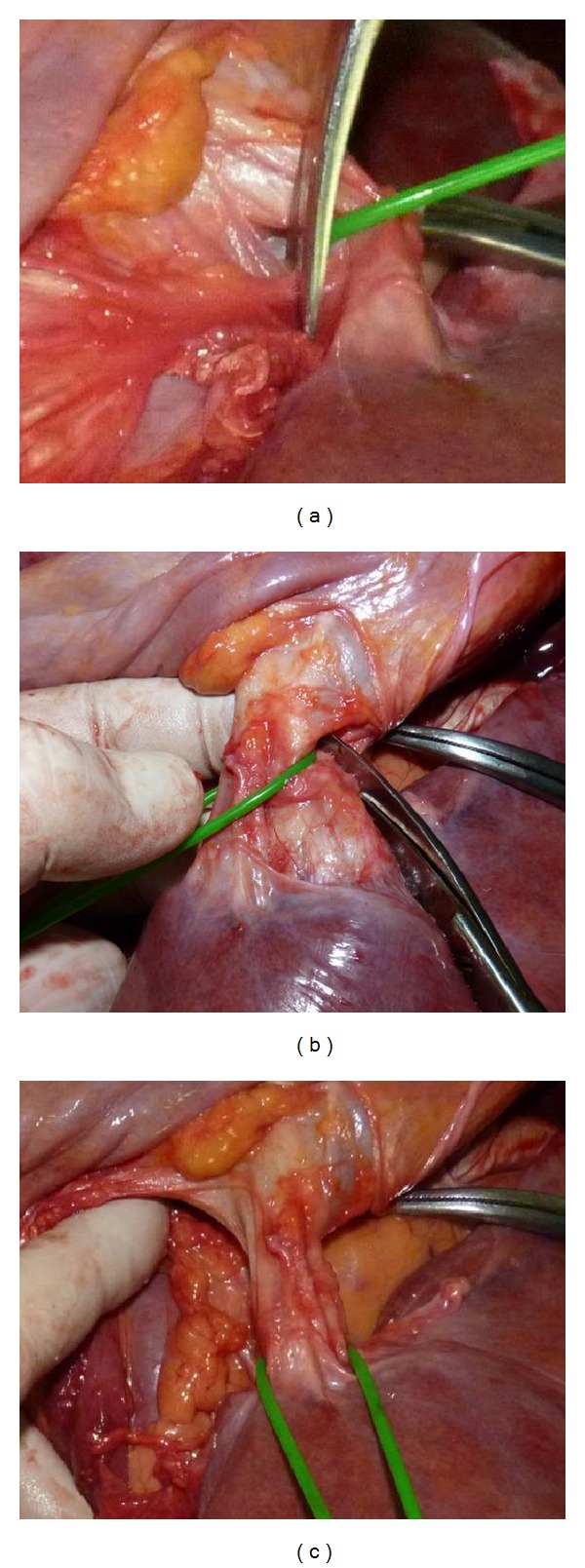
((a), (b)) Steps ofcircular diaphragmotomy; (c) the supradiaphragmatic IVC and the right atrium after circular diaphragmotomy.

**Figure 5:**
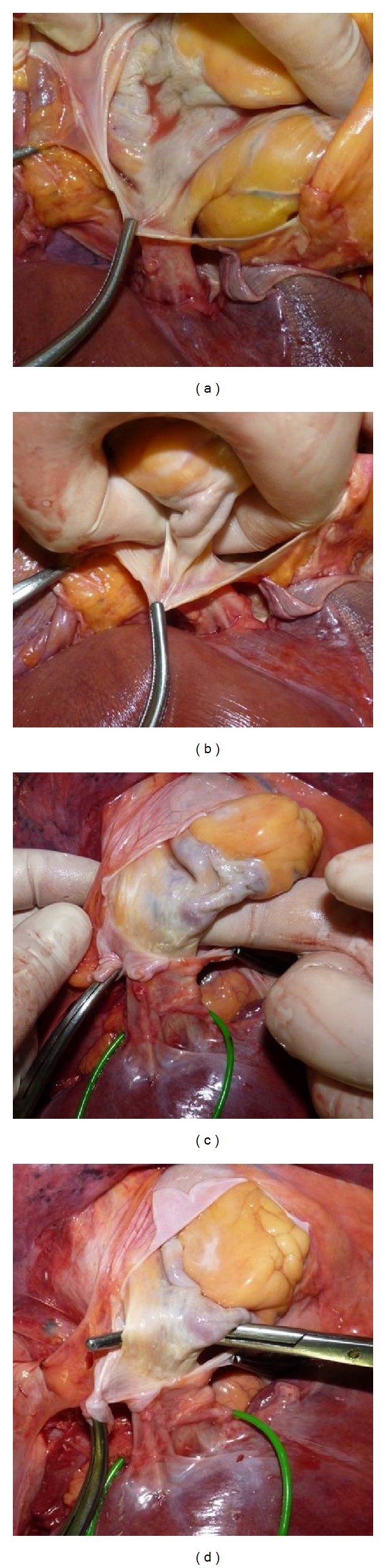
(a) Pericardial cavity opened; ((b), (c)) palpation of the intrapericardial IVC; (d) surgical clamp passed posterior to the intrapericardial IVC through the pericardial layers.

After transverse diaphragmotomy we attempted to pass the tourniquet around the supradiaphragmatic inferior vena cava (Figure 2). Afterwards, the access was extended by an additional perpendicular incision (T-shaped diaphragmotomy) and the tourniquet was passed around the cavoatrial junction area (Figure 3). The next stage involved complete separation of the inferior vena cava from the diaphragm (circular diaphragmotomy) and external palpation of the supradiaphragmatic IVC and the right atrium (Figure 4). Only after this, the longitudinal incision was applied to open the pericardium; then followed the analysis of the length between the visceral pericardial layers, comprising the intrapericardial IVC, and the ability to pass the tourniquet around the vena cava at this level (Figure 5).

The ability to perform each of these steps was evaluated using the following scale: easy (100 points), difficult (50 points), and impossible (0 points). With respect to the risk level the intervention was graded as safe (100 points), risky (50 points), and that which caused the injury of vessels or the right phrenic nerve (0 points).

At the final stage of the examination the entire length of the inferior vena cava was dissected longitudinally. Then the length and width of the supradiaphragmatic IVC as well as the size and topography of the phrenic vein insertions were assessed.

## 3. Results

In our study the average length of the supradiaphragmatic inferior vena cava (from the right atrial appendage to the diaphragm) was 20.6 mm (10 to 35 mm) and the width was 28.7 mm (22 to 35 mm).

The insertions of the phrenic veins at the level of the supradiaphragmatic IVC were identified only in 4 (11.4%) of 35 patients. Their number varied from 1 to 2. The average diameter of these vessels did not exceed 2.0 mm (1.0 to 3.0 mm). The insertions of the phrenic veins were localized mainly on the right side of the anterior and anterolateral half circle of the supradiaphragmatic inferior vena cava at the 2, 3, 9, and 10 o'clock positions (Table 1). We found no draining in the veins on the posterior half circle of the IVC in any of the cases.

In most cases, the diaphragmatic veins drain into the IVC at the level of the diaphragm or below. Their total number in 35 patients was 108. The insertions of these vessels were mainly localized on the anterior half circle of the IVC, with average diameter 2.6 mm (1.0 to 6.0 mm), and well visualized during diaphragmotomy. The inflow of the phrenic veins on the posterior half circle of the IVC was much less frequent. The vein insertions also prevailed on the right side. The damage to the phrenic veins was mostly observed during circular (33 (94.3%) of 35 cases) and transverse (8 (22.9%) of 35 cases) diaphragmotomy. However, providing hemostasis in such situation generally does not pose any problem, given the small diameter and “convenient” localization of these blood vessels.

It was possible to identify the right phrenic nerve in 33 (94.3%) of 35 patients. The nerve with its branches was visualized only from the chest aspect. It was located on the posterolateral fibrous pericardium and penetrated into the diaphragm slightly more to the right of the inferior vena cava opening (at the 7-8 o'clock position). In most cases, the nerve was surrounded by loose fatty tissue and could be easily detached 7–10 mm from the line of fusion of the pericardium and the diaphragm. Five patients had an abdominal branch of the right phrenic nerve passing through the diaphragm immediately next to the right wall of the intrapericardial inferior vena cava.

Damage to the large diaphragmatic branches and the main trunk of the right phrenic nerve was not detected in any of the cases. Injury to the abdominal branch of the right phrenic nerve occurred in 3 (8.6%) patients (transverse diaphragmotomy-2, circular diaphragmotomy-1).

Feasibility and safety of different approaches regarding the ability to pass the tourniquet around the supradiaphragmatic section of the inferior vena cava are shown in Tables 2, 3, and 4.

The results of our work have demonstrated that the isolation of the supradiaphragmatic IVC and cavoatrial junction was most easily performed through T-shaped and circular diaphragmotomy (grade “easy” was registered in 74.3% and 80% of the patients, resp., as compared to 31.4% for transverse diaphragmotomy and 40% for the IVC isolation in the pericardial cavity). In 4 (11.4%) cases when using transverse diaphragmotomy and in 2 (5.7%) cases of attempt to isolate the IVC in the pericardial cavity we failed to pass the tourniquet around the supradiaphragmatic inferior vena cava.

The risk analysis has demonstrated the greatest safety level for T-shaped diaphragmotomy (grade “safe” was recorded in 60% of cases). The intervention through transverse diaphragmotomy, circular diaphragmotomy, and isolation of the IVC in the pericardial cavity was graded as “risky” in 80%, 62.9%, and 82.9% of cases, respectively.

## 4. Discussion

The use of cardiopulmonary bypass and circulatory arrest during surgery for tumor thrombi of the inferior vena cava extending above the diaphragm, in some cases, is accompanied by serious complications (coagulopathy, neurological disorders, and multisystem failure). This approach significantly increases trauma level and duration of the operation (the set-up time for cardiopulmonary bypass is at least 30 minutes). On the other hand, one should take into account that the use of this technique requires performing median sternotomy, which also significantly increases the time of operation and can result in serious postoperative complications. Therefore, at present many surgeons tend to search for alternative approaches that could help in removing the thrombi in the supradiaphragmatic vena cava without sternotomy, cardiopulmonary bypass, and circulatory arrest.

Exposure of the supradiaphragmatic inferior vena cava and the right atrium is one of the most important stages during removal of the tumor thrombi extending above the insertions of the major hepatic veins. In recent years several reports have been published relating to the approach to the intrapericardial inferior vena cava through the diaphragm from the abdominal cavity (Table 5).

**Figure 6:**
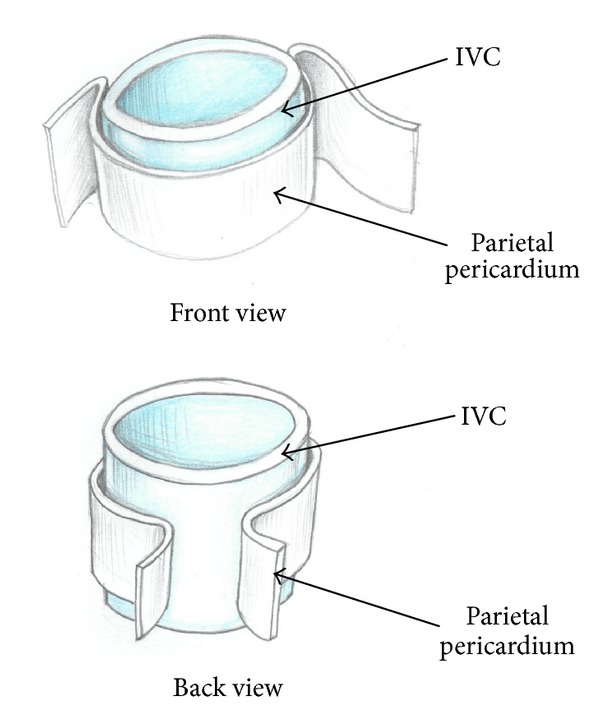
Relation between the parietal pericardium and the intrapericardial IVC.

Most of surgeons describe the use of anterior longitudinal or transverse diaphragmotomy and pericardiotomy with isolation of the IVC in the pericardial cavity [3, 8–10]. Chen et al. proposed forming a window in the diaphragm and pericardium [6]. Despite the distinct advantages of this approach (direct access to the right atrium and intrapericardial segment of the inferior vena cava, the minimum probability of damage to the phrenic veins and right phrenic nerve), there are certain problems associated with its use. They can be explained by anatomical features of the supradiaphragmatic inferior vena cava, which after passing through the tendon center of the diaphragm deviates slightly posteriorly. In this regard, the access to the intrapericardial IVC via anterior longitudinal diaphragmotomy and pericardiotomy is deep and not convenient enough. Besides, the intrapericardial IVC is not completely surrounded by the parietal pericardium, but enveloped by it laterally and anteriorly. The posterior vena cava is usually located extrapericardially at this level. In this case, the pericardial layers form a kind of mesenterium having different thicknesses in different patients (Figure 6). Therefore, passing the tourniquet around the intrapericardial IVC requires perforation of both layers of the pericardium. This maneuver is insecure due to possible damage to the posterior wall of the inferior vena cava and uncontrolled bleeding, since the instrument is passed through in a blind manner. Another limitation of transpericardial approach is inability to manually displace the tumor thrombus below the diaphragm.

It also should be kept in mind that opening the pericardium during the operation can cause raising the right ventricular end-diastolic and end-systolic pressure that may result in decreased cardiac output [11]. There have been multiple reports describing development of constrictive or purulent pericarditis as well as cardiac tamponade after pericardiotomy in the postoperative period [12]. In this connection, alternative approaches to the supradiaphragmatic IVC without opening the pericardium have been developed.

Ciancio and Soloway proposed circular detachment of the inferior vena cava from the tendon center of the diaphragm without opening the pericardium [5]. Through this approach the authors performed removal of the tumor thrombus penetrating into the right atrium. Its main advantages are preserving the pericardium integrity, good control of the intrapericardial inferior vena cava, and ability to manually displace the tumor thrombus downwards. However, the circular diaphragmotomy may be accompanied by damage to the right phrenic nerve and diaphragmatic veins. To reduce the risk of right phrenic nerve injury the authors separate the IVC from the tendon center of the diaphragm immediately in the area of their fusion, but this maneuver may have high probability of vena cava injury.

Mizuno et al. described the procedure of wide longitudinal diaphragmotomy without opening the pericardium [7]. The authors report that this approach has low injury level and is easy to perform.

However, the summarized experience of surgical interventions made via the described approaches is inadequate being mainly reports about single operations. Besides, no detailed study on the anatomy of the intrapericardial inferior vena cava and the caval opening of diaphragm in terms of vena cava thrombectomy has been performed.

We have studied feasibility and safety of intrapericardial IVC isolation via four different approaches: transverse, T-shaped, circular diaphragmotomy, and through the pericardial cavity. These approaches can be regarded as independent options of surgical access to the supradiaphragmatic IVC, and on the other hand, they can be successive steps of intrusion to the mediastinum through the abdominal cavity.

The results of our study have demonstrated that the diaphragmatic veins rarely drain into the supradiaphragmatic inferior vena cava (6.1%). Moreover, all the diaphragmatic veins enter the supradiaphragmatic IVC exclusively on its anterior half circle. This data is similar to the results obtained by Birincioglu et al. [13]. They demonstrate relative safety in terms of possible damage to the phrenic veins for longitudinal approaches to the supradiaphragmatic IVC either with opening of the pericardium or without. Transverse and T-shaped approaches as well as circular diaphragmotomy more often can result in damage to the phrenic veins being drained at the level of the diaphragm and subdiaphragmatic IVC. However, we do not regard damage to the phrenic veins as a serious surgical problem, given their small diameter and “convenient” anterior location in most patients.

None of the observations in our study have detected any damage to the large diaphragmatic branches and the main trunk of the right phrenic nerve. Injury to the abdominal branch of the right phrenic nerve occurred in 3 (8.6%) patients (transverse diaphragmotomy-2, circular diaphragmotomy-1).

Injury to the right phrenic nerve can cause serious problems, including paralysis of the right hemidiaphragm and respiratory failure, which is an extremely severe complication of any surgical intervention in the suprahepatic inferior vena cava [14]. In particular, paralysis of the right hemisphere of the diaphragm is quite common when the classic technique of orthotopic liver transplantation is used. McAlister et al. discovered that after liver transplantation the right phrenic nerve injury and paralysis of the right hemidiaphragm occurred in 79% and 38% of 48 patients, respectively [15].

Some surgeons recommend thorough examination of the diaphragm surface prior to diaphragmotomy in order to identify the phrenic nerve branches, and they consider this as a base of prevention of n. phrenicus dexter injury. We are extremely skeptical about this recommendation, since in our study we could visually identify the phrenic nerve and its branches only from the chest aspect. At examining the diaphragmatic surface from the aspect of the abdominal cavity we could not identify the branches of this nerve in any of our observations.

Our opinion is that the chance of n. phrenicus dexter injury is minimal with the use of T-shaped diaphragmotomy or isolation of the IVC through the pericardial cavity. We have not found a single case of injury to the main trunk or large diaphragmatic branches of this nerve. Only abdominal branches, having no serious impact on the function of the diaphragm, were injured. However, to confirm the data, we need to carry out an extensive clinical investigation of the diaphragm function after surgical removal of the “high” tumor thrombus.

Based on the experience gained, we suggest the following recommendations for prevention of injury of the right phrenic nerve.

**Figure 7:**
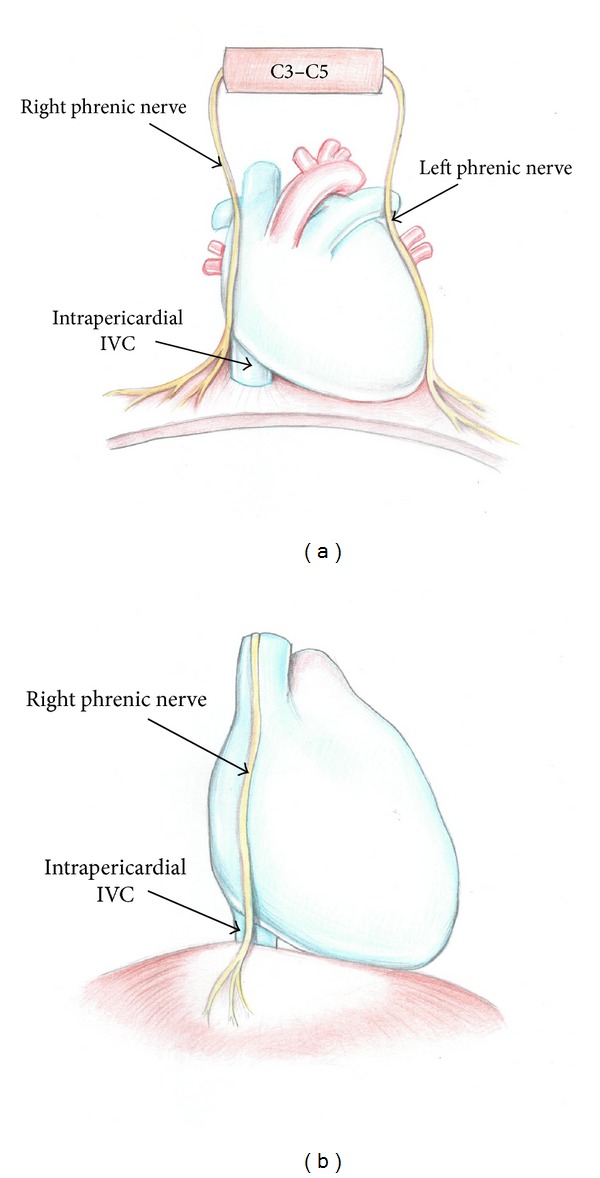
Anatomy of the right phrenic nerve ((a) front view; (b) side view).

**Figure 8:**
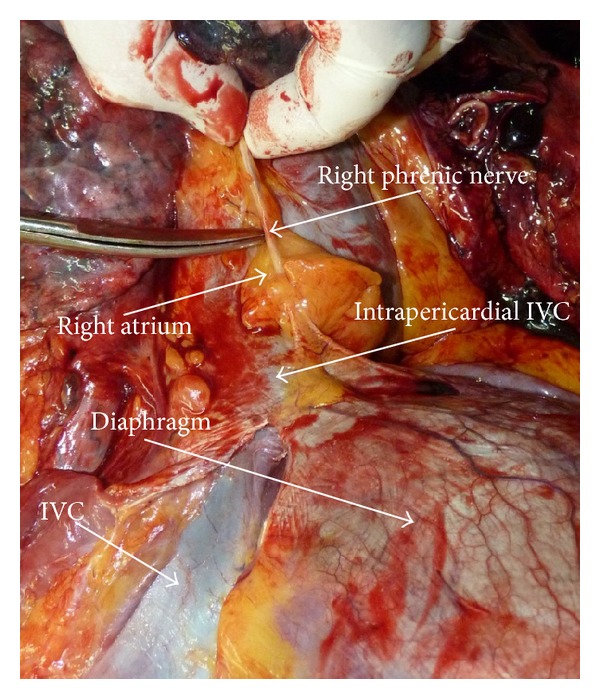
The right phrenic nerve; autopsy case; back view.

1. The most “harmful,” in terms of injury to the phrenic branches or the main trunk of the right phrenic nerve, are apparently transverse and circular diaphragmotomy.
2. Taking into account that the most problematic area in terms of possible nerve damage is the right lateral and posterolateral surface of the inferior vena cava, the diaphragm in this area during transverse or circular diaphragmotomy should be dissected as close as possible to the vena cava. In the other areas cutting the diaphragm can be made 3–5 mm away from the surface of the vein (Figures 7 and 8).
3. T-shaped diaphragmotomy allows passing the tourniquet around the cavoatrial segment without opening the pericardium and is not accompanied by the risk of damage to the phrenic vessels. We believe that circular diaphragmotomy is necessary only when manual displacement of the thrombus apex below the diaphragm level is used.
4. After cutting the diaphragm you should separate the right surface of the supradiaphragmatic inferior vena cava from the fatty tissue very carefully. It is just in this area where the right phrenic nerve transfers from the fibrous pericardium to the diaphragm surface. The amount of fatty tissue that surrounds the nerve is extremely individual, but in most cases, this fatty tissue is well defined, which helps to move the nerve within 7–10 mm. Therefore, the dissection of the supradiaphragmatic IVC should be performed very carefully and as close as possible to the right wall of the vein. This technique also will help in avoiding opening the right pleural cavity during surgery.
5. Given that the tendon center of the diaphragm has quite thin and mobile structure, a surgeon in some cases may be tempted to place a clamp on the IVC together with the diaphragm without isolation of the supradiaphragmatic vena cava. We are strongly against such an approach, as in this case the phrenic nerve injury is inevitable and the risk of clamp slippage is high due to the large mass of tissue clamped therein.

## 5. Conclusion

Surgical access to the right atrium and supradiaphragmatic IVC through the diaphragm from the abdominal cavity is an adequate alternative to sternotomy. A chance of damage to the main trunk of the right phrenic nerve during this approach is quite low. From our point of view, the most secure and easy-to-perform access for isolation of the supradiaphragmatic IVC through the abdominal cavity is T-shaped diaphragmotomy without opening the pericardium.

## Figures and Tables

**Table 1:** Localization of the phrenic vein insertions.

IVC level	Localization of insertions									
	The right anterior half circle of the IVC		The left anterior half circle of the IVC		The right posterior half circle of the IVC		The left posterior half circle of the IVC		Total	
	(*N*)	(%)	(*N*)	(%)	(*N*)	(%)	(*N*)	(%)	(*N*)	(%)
The supradiaphragmatic IVC	5	4.3	2	1.8	0	0	0	0	7	6.1
The fusion of the IVC with the diaphragm and below the diaphragm	58	50.4	42	36.5	7	6.1	1	0.9	108	93.9

Total	63	54.8	44	38.3	7	6.1	1	0.9	115	100

**Table 2:** Feasibility analysis of various approaches to the supradiaphragmatic IVC.

Feasibility	Easy		Difficult		Impossible		Average (points)
	(*N*)	(%)	(*N*)	(%)	(*N*)	(%)	
Transverse diaphragmotomy	11	31.4	20	57.2	4	11.4	60
T-shaped diaphragmotomy	26	74.3	9	25.7	0	0	87.1
Circular diaphragmotomy	28	80	7	35	0	0	90
IVC isolation in the pericardium	14	40	19	54.3	2	5.7	67.1

**Table 3:** Safety analysis of different approaches to the supradiaphragmatic IVC.

Safety	Safe		Risky		Trauma		Average (points)
	(*N*)	(%)	(*N*)	(%)	(*N*)	(%)	
Transverse diaphragmotomy	4	11.4	28	80	3	8.6	51.4
T-shaped diaphragmotomy	21	60	14	40	0	0	80
Circular diaphragmotomy	11	31.4	22	62.9	2	5.7	62.9
IVC isolation in the pericardium	6	17.1	29	82.9	0	0	58.6

**Table 4:** Feasibility options and risk analysis of different approaches to the supradiaphragmatic IVC.

Combined parameters	Type of diaphragmotomy							
	Transverse diaphragmotomy		T-shaped diaphragmotomy		Circular diaphragmotomy		IVC isolation in pericardium	
	(*N*)	(%)	(*N*)	(%)	(*N*)	(%)	(*N*)	(%)
Easy + safe	2	5.7	19	54.3	9	25.7	3	8.6
Easy + risky	9	25.7	7	20	18	51.4	11	31.4
Easy + trauma	0	0	0	0	1	2.9	0	0
Difficult + safe	2	5.7	2	5.7	2	5.7	3	8.6
Difficult + risky	15	42.9	7	20	4	11.4	16	45.7
Difficult + trauma	3	8.6	0	0	1	2.9	0	0
Impossible + risky	4	11.4	0	0	0	0	2	5.7
Difficult + trauma	0	0	0	0	0	0	0	0

Total	35	100	35	100	35	100	35	100

**Table 5:** Summary of published data on the use of different approaches to the supradiaphragmatic IVC through the abdominal cavity.

Authors	Position as to the pericardial cavity	Type of incision in the central tendon of the diaphragm
Davydov et al. [3]	Through the pericardium	Anterior longitudinal
Belgrano et al. [10]	Through the pericardium	Anterior longitudinal
Bassi et al. [8]	Through the pericardium	Anterior longitudinal
Ciancio and Soloway [5]	Outside the pericardium	Circular
Chen et al. [6]	Through the pericardium	A window in the diaphragm
Facciuto et al. [9]	Through the pericardium	Anterior longitudinal
Mizuno et al. [7]	Outside the pericardium	Anterior longitudinal
Miyazaki et al. [4]	Through the pericardium	Anterior transverse
